# Data from acellular human heart matrix

**DOI:** 10.1016/j.dib.2016.04.069

**Published:** 2016-05-18

**Authors:** Pedro L Sánchez, Mª Eugenia Fernández-Santos, Mª Angeles Espinosa, Mª Angeles González-Nicolas, Judith R Acebes, Salvatore Costanza, Isabel Moscoso, Hugo Rodríguez, Julio García, Jesús Romero, Stefan M Kren, Javier Bermejo, Raquel Yotti, Candelas Pérez del Villar, Ricardo Sanz-Ruiz, Jaime Elizaga, Doris A Taylor, Francisco Fernández-Avilés

**Affiliations:** a Department of Cardiology, Hospital General Universitario Gregorio Marañón, Universidad Complutense de Madrid, Instituto de Investigación Sanitaria Gregorio Marañon (IiSGM), Madrid, Spain; b Bioartifical Organs Laboratory, Instituto de Investigación Sanitaria Gregorio Marañon (IiSGM), Madrid, Spain; c Hospital Universitario de Salamanca, IBSAL, Salamanca, Spain; d Cell Production Unit, Instituto de Investigación Sanitaria Gregorio Marañon (IiSGM), Madrid, Spain; e Department of Cardiovascular Development and Repair, Centro Nacional de Investigaciones Cardiovasculares Carlos III (CNIC), Spain; f Department of Cardiac Surgery, Hospital General Universitario Gregorio Marañón. Universidad Complutense de Madrid, Instituto de Investigación Sanitaria Gregorio Marañon (IiSGM), Madrid, Spain; g Department of Pathology, Hospital General Universitario Gregorio Marañón, Universidad Complutense de Madrid. Instituto de Investigación Sanitaria Gregorio Marañon (IiSGM), Madrid, Spain; h Photography & Comunication, Hospital General Universitario Gregorio Marañón, Universidad Complutense de Madrid. Instituto de Investigación Sanitaria Gregorio Marañon (IiSGM), Madrid, Spain; i Center for Cardiovascular Repair, University of Minnesota, Minneapolis, USA; j Regenerative Medicine Research, Texas Heart Institute, Houston, USA

## Abstract

Perfusion decellularization of cadaveric hearts removes cells and generates a cell-free extracellular matrix scaffold containing acellular vascular conduits, which are theoretically sufficient to perfuse and support tissue-engineered heart constructs. This article contains additional data of our experience decellularizing and testing structural integrity and composition of a large series of human hearts, “Acellular human heart matrix: a critical step toward whole heat grafts” (Sanchez et al., 2015) [1]. Here we provide the information about the heart decellularization technique, the valve competence evaluation of the decellularized scaffolds, the integrity evaluation of epicardial and myocardial coronary circulation, the pressure volume measurements, the primers used to assess cardiac muscle gene expression and, the characteristics of donors, donor hearts, scaffolds and perfusion decellularization process.

**Specifications Table**

**Table:** 

Subject area	*Biology*
More specific subject area	*Bioengineering human heart matrix*
Type of data	*Table, image, text file, figure*
How data was acquired	*Echocardiography (General Electric),* linear mixed-effects models (LME, S-Plus version 8.0, Tibco Software) and angiography (Siemens)
Data format	*Analyzed, processed*
Experimental factors	*Human hearts used in the study were not suitable for transplantation.*
Experimental features	*Heart decellularization perfusion was performed to remove cells but retain the extracellular matrix scaffold. Characteristics of the scaffold valves, chambers and vasculature were assessed using echocardiography, pressure-volume measurements and coronary angiography. The effect of the human scaffold on the differentiation of human cardiac progenitor cells was also analyzed with different primers*
Data source location	*Madrid, Spain*
Data accessibility	*Within this article*

**Value of the data**

• The data provides the schematic information of a decellularization heart perfusion technique that could be followed as a standardized technique for additional decellularization studies.
• The data provides the detail information of the characteristics of donors and heart scaffolds. These physiologic data will provide researchers with important age- and sex-specific reference ranges for evaluating experimental results.
• It also provides the basis of different experiments for a clear demonstration of valve competence, coronary angiography assessment and pressure-volume measurements. These novel assays could be useful tools for the in vitro evaluation of decellularized heart scaffolds.
• The data provides the primers used to assess cardiac gene expression in human cardiac progenitor cells grown on human decellularized extracellular matrices. The primers profile data could be used to identify cardiac cell differentiation.

## 1 Data

Dataset provided in this article shows the perfusion decellularization protocol we used to remove the cells from 39 human hearts while retaining the extracellular matrix [1]. The characteristics of the decellularized valves and anatomical aspects of the decellularized cardiac vessels were assessed by using echocardiography and coronary angiography, respectively. In addition, the passive pressure-volume relationship of the left and right ventricle was measured in 8 human hearts before and after decellularization. Finally, primers used to assess cardiac gene expression in human cardiac progenitor cells grown on human decellularized extracellular matrix are described.

## 2 Experimental design, materials and methods

### 2.1 Heart decellularization technique

**Fig. 1:**
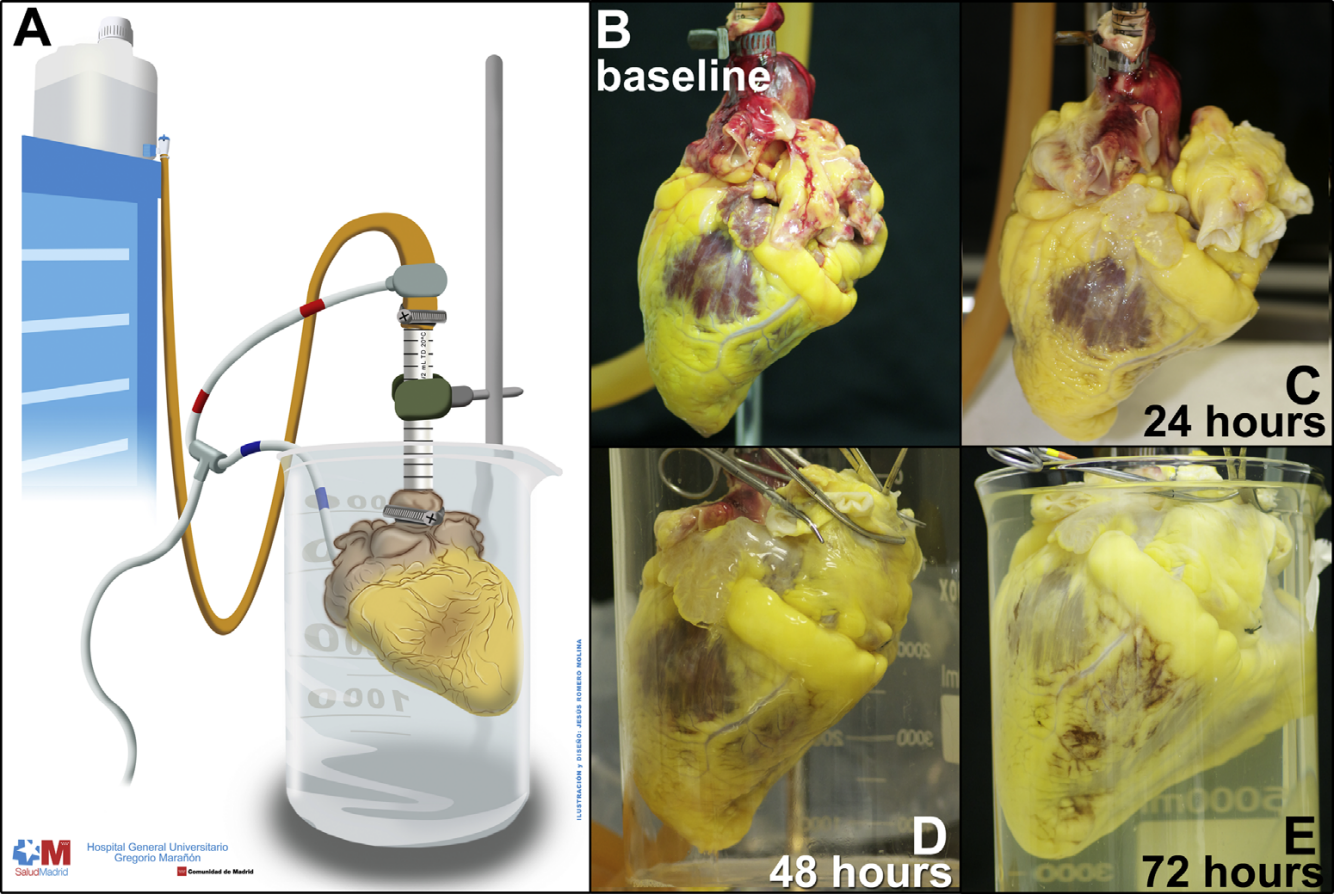
Heart decellularization technique.

To remove cells but retain the extracellular matrix (ECM) we applied our previously described perfusion decellularization technique, [2] with 1% sodium dodecyl sulfate (SDS) detergent in deionized water via antegrade coronary perfusion of the ascending aorta. Schematic of perfusion decellularization of human heart is shown in Fig. 1, Panel A. Perfusion pressure is monitored to perfuse the heart at ~80–100 mm Hg (normal blood pressure) and is changed by altering the height of the dispensing container that feeds the decellularization or washing reagents into the aorta. During the decellularization period (Fig. 1, Panels B to E) over 72–96 h, there is a loss of color as decellularization proceeds first from the thinnest regions (great vessels and atria) and progresses toward the thickest areas of the heart so that the decellularized parts become more translucent and devoid of most color.

### 2.2 Characteristics of donors and heart scaffolds

Characteristics of donors, donor hearts, scaffolds and perfusion decellularization process are shown in Table 1. Thirty-nine human hearts were decellularized and 13 human hearts were used as controls.

### 2.3 Valve competence evaluation

**Fig. 2:**
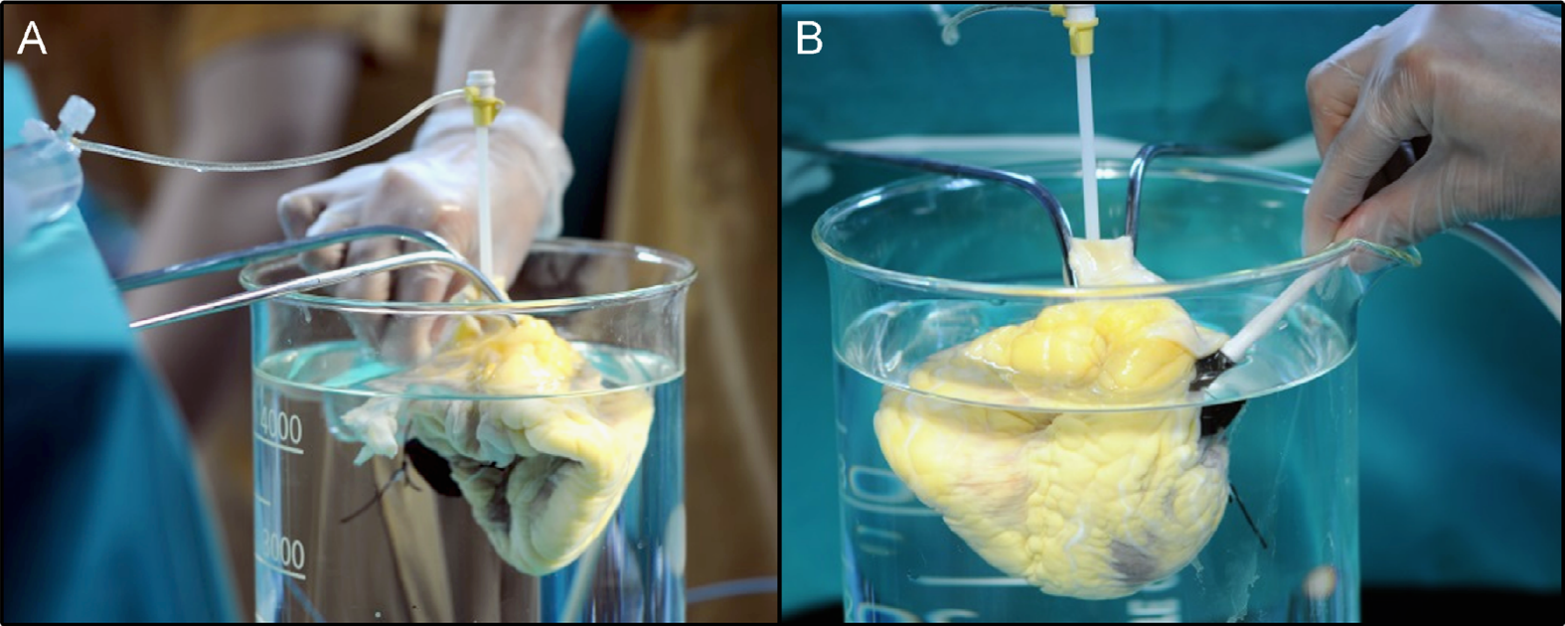
Valve competence evaluation during systole and diastole using echocardiography from the epicardium.

Macroscopic valve inspection was performed by cardiac surgeons and by echocardiography. Valve obstruction was easily excluded by macroscopic valve inspection of the valves. The semilunar valves (pulmonary and aortic) prevented retrograde flow back into the ventricles by a simply maneuver of filling with saline the ascending aorta and the pulmonary arteries. The atrioventricular valves (tricuspid and mitral) were evaluated with echocardiography filling the LV and RV through a 5-French intravascular sheath, inserted across the hermetically closed sutured aortic or pulmonary valves, with isotonic saline using 60 ml syringes (Fig. 2). Systole and diastole was modified by aspiration or injection of saline through the syringes helped by an external compression of the ventricles with the hand. During echocardiography evaluation, the scaffolds were submerged in deionized water. We obtained images with a broadband 14 MHz transducer (General Electric).

### 2.4 Integrity of epicardial and myocardial coronary circulation

**Fig. 3:**
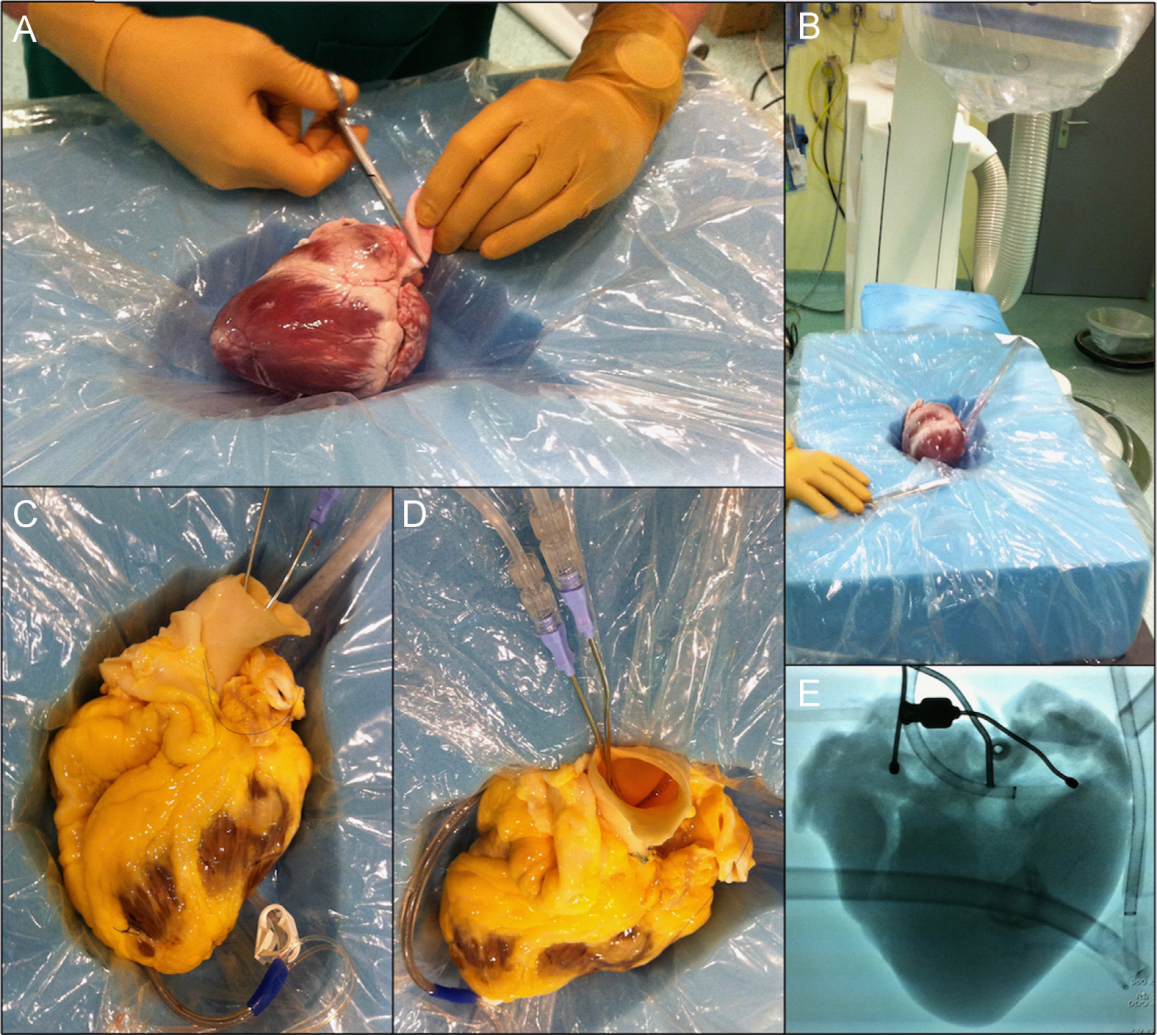
Angiographic coronary vascular study.

Simultaneous angiography of the left and right coronary artery was assed to test the integrity of epicardial and myocardial coronary circulation of the whole decellularized human hearts. Controls and decellularized hearts were positioned in a foam mold, with a similar height to the anteroposterior thorax length, where the heart shape was dug (Fig. 3, Panel A and B). To record the coronary angiographic sequences we used an Artis Zee biplane system (Siemens Healthcare) (Fig. 3, Panel B). Both coronary ostium were cannulated with a conventional irrigating DeBakey heparing cannula (3 mm tip, 2 ½", 6.3 cm length, Pilling) and simultaneous angiography injections were performed using a conventional mechanical injector pump (Fig. 3, Panel C-D). We also cannulated the coronary sinus with a conventional retrograde cannula (Medtronic) in order to simultaneously drain the injected contrast media (iodixanol, Visipaque^TM^, General Electric).

### 2.5 Pressure–volume measurements

**Fig. 4:**
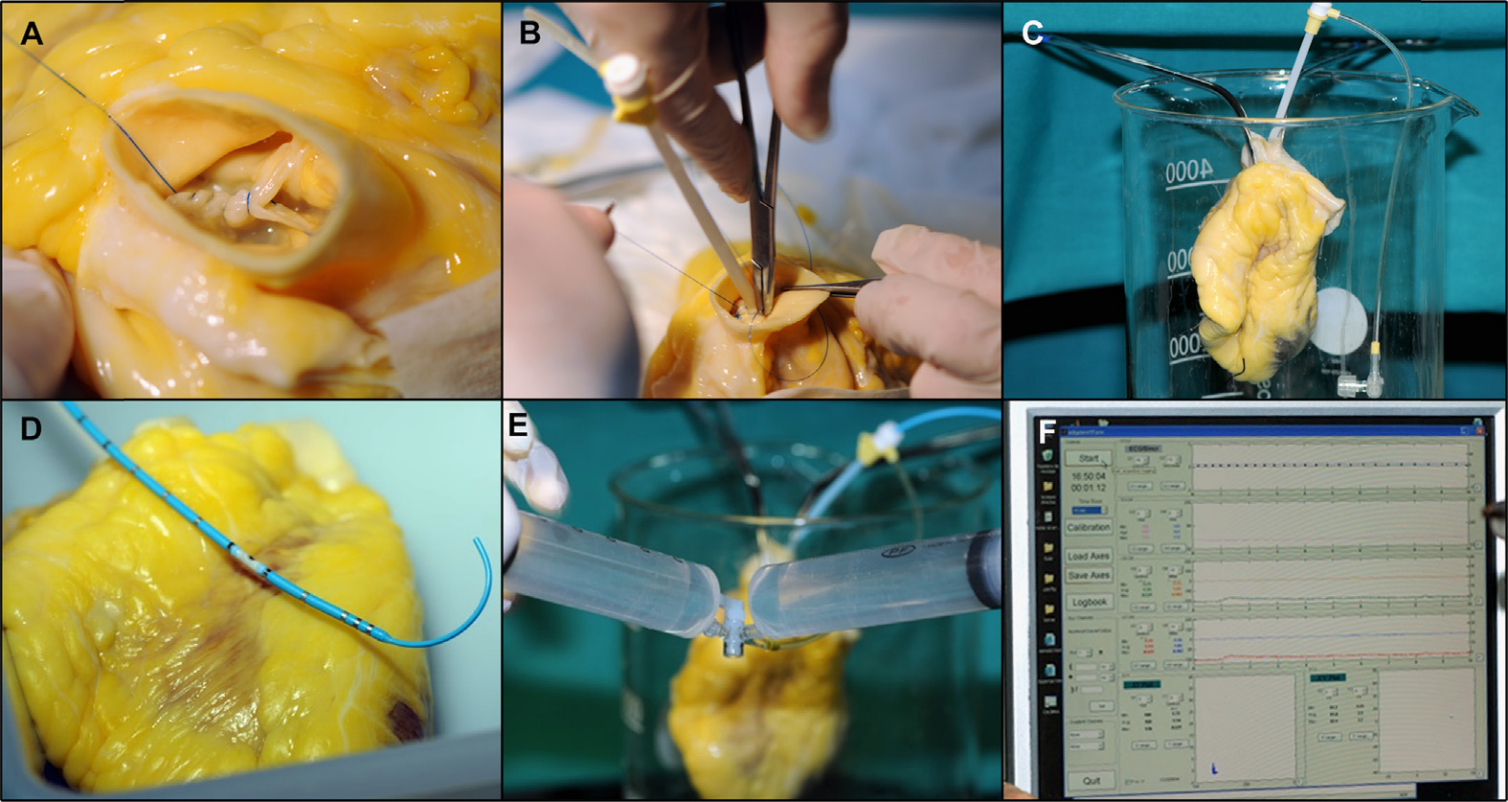
Pressure–volume measurement to assess global chamber mechanical passive properties.

We studied the relative contributions of the ECM to overall passive mechanical global properties of the left ventricle (LV) and right ventricle (RV) chambers. Mechanical properties of the decellularized human hearts were compared to the intact heart before decellularization both for the LV and RV, slightly modifying previously reported methods. [3], [4], [5] LV and RV are hermetically closed suturing aortic (Fig. 4, Panel A), pulmonary, mitral and tricuspid valves in a fully closed position. A 5-French intravascular sheath is inserted across the aortic (Fig. 4, Panel B) and pulmonary valve depending whether LV or RV pressure-volume measurements are calculated. Non- compliant polyethylene balloons are inserted across the aortic or pulmonary valves and hermetically attached to the 5-French intravascular sheath because saline leakage was observed in scaffolds (Fig. 4, panel C). A 5-French high-fidelity micromanometer (Millar-Instrument) was placed inside the balloon and connected to a 3-port manifold (Fig. 4, Panel D). Pressure and volume are then modified by aspiration followed by slow injection of isotonic saline using 60 ml syringes attached to the manifold (Fig. 4, Panel E). Differences in repated measures obtained during pre- and post-decellularization of each specimen are assessed by linear mixed-effects models (LME, S-Plus version 8.0, Tibco Software)(Fig. 2, Panel F).

### 2.6 Cardiac gene expression

Primers used to assess cardiac gene expression in human cardiac progenitor cells grown on human decellularized ECM for 21 days are shown in Table 2.

### 2.7 Summarizing table

Table 3 summarizes critical steps and troubleshooting, number of samples analyzed and homogeneity results of the aforementioned principal protocols.

## Figures and Tables

**Table 1:** Characteristics of donors, donor hearts, scaffolds, and perfusion decellularization process.

**Sex**	**Age**	**Heart weight**	**Scaffold weight**	**Time to start of decellularization**	**Days of decellularization**
Male	50	558	452	9 h	4
Male	73	466	436	12 h	7
Female	58	332	224	24 h	4
Female	50	369	292	24 h	7
Male	80	398	327	24 h	7
Female	67	488	457	24 h	8
Female	87	358	Control	Control	Control
Female	35	267	Control	Control	Control
Female	54	587	522	9 h	7
Male	59	550	500	13 h	8
Male	49	380	351	8 h	4
Male	76	343	288	24 h	4
Female	68	526	432	3 days	5
Female	65	314	283	3 days	4
Female	17	231	189	8 h	5
Male	57	671	657	5 h	5
Male	66	435	344	24 h	4
Male	61	512	Control	Control	Control
Male	41	398	Control	Control	Control
Male	41	680	572	12 h	7
Female	72	284	Control	Control	Control
Male	43	353	300	24 h	4
Male	58	376	331	4 days	4
Male	59	432	376	24 h	4
Male	57	326	Control	Control	Control
Male	46	358	Control	Control	Control
Male	30	291	Control	Control	Control
Male	78	298	Control	Control	Control
Male	70	644	528	10 h	6
Male	78	430	354	5 days	4
Male	55	252	214	12 h	7
Female	32	271	171	12 h	3
Male	56	303	249	2 days	7
Female	60	404	334	60 h	5
Female	66	335	Control	Control	Control
Female	80	464	361	12 h	7
Male	58	419	279	10 h	6
Male	73	515	439	12 h	4
Male	52	457	435	18 h	8
Male	70	464	393	12 h	8
Male	52	565	373	24 h	6
Male	54	362	Control	Control	Control
Male	59	410	Control	Control	Control
Male	67	373	306	12 h	8
Female	64	305	244	12 h	8
Female	64	321	238	18 h	8
Female	63	526	418	18 h	4
Female	38	343	Control	Control	Control
Female	56	265	214	15 h	8
Female	65	327	295	18 h	8
Male	52	682	532	12 h	8
Male	70	677	525	12 h	8

**Table 2:** Primers used to assess cardiac muscle gene expression in hCPCs grown on human dECM for 21 days.

**Gene**	**Sense**	**Anti-Sense**
bMHC	5′-GCTGGGGCTGATGCGCCTAT-3′	5′-TGGGGATGGGTGGAGCGCAA-3′
cActin	5′-GACGAGGAGACCACCGCCCT-3′	5′-ACCATAACTCCCTGGTGCCGC-3′
GATA4	5′-AGCAGCTTCTGCGCCTGTGG-3′	5′-TGGGGGCAGAAGACGGAGGG-3′
MEF2C	5′- TCCTGCAAATATGGCCCTAG-3′	5′- CCTGACACACCGGGATTGTT-3′
Nkx2.5	5′-TCACCGGCCAAGTGTGCGTC-3′	5′-GCAGCGCGCACAGCTCTTTC-3′
Troponin T	5′-GGAGCAGGAAGAAGCAGCTGTTGA-3′	5′-TTCTGCCCTGGTCTCCTCGGTC-3′
GusB	5′-CAACGAGCCTGCGTCCCACC-3′	5′-ACGGAGCCCCCTTGTCTGCT-3′

**Table 3:** Critical steps and troubleshooting of decellularization technique and valve competence, coronary circulation and pressure-volume assessments.

	**Principal steps**	**Trouble shooting**	**Samples**	**Homogeneity**
**Decellularization technique**	• Perfusion decellularization is performed with 1% sodium dodecyl sulfate (SDS) detergent in deionized water via antegrade coronary perfusion through the ascending aorta. Approximately 60 l of solution, during 4 days perfusion, is needed to decelullarize a human heart. • After SDS perfusion extensive rinsing with approximately 20 l of water followed by 10 l of penicillin-streptomycin-containing phosphate-buffered saline (PBS) is performed.	• The quality of SDS powder varies between brands. SDS solution should be completely clear without aggregates. • Pressure should be monitorized in order to avoid leaks through the translucent wall chamber because of overpressure. • Yellow-brown lipofuscin precipitates are observed within decellularized myocardium if overpressure during antegrade coronary perfusion	N=39	Yes
**Valve competence**	• Competency of valves was assed by macroscopic inspection, by filling with saline the ascending aorta and pulmonary arteries in the case of the semilunar valves (aortic and pulmonary), and by echocardiography.	• To obtain adequate echocardiography images make sure the heart scaffold is fully submerged in 36 °C deionized water.	N=5	Yes
**Coronary circulation**	• Left and right coronary ostium are cannulated with a conventional cardiac surgery irrigating cannula. In our cases we used a Debakey 3 mm cannula. • The coronary sinus is also cannulated with a conventional retrograde cannula in order to drain the injected contrast media.	• An automatic contract injection was preferred over a manual standard technique as it allowed us to control of injection rate and amount of contrast to be delivered. • Angiographic runs had to be long enough to allow filling of the venous coronary system.	N=5	Yes
**Pressure-volume assesment**	• Ensure LV and RV are hermetically closed suturing all four cardiac valves in a fully closed position. • Soak the PV conductance catheter in a warm 36 °C saline for 30 min before measurements • Pressure and volume are modified by aspiration, followed by slow injection of isotonic saline with the use of 60 ml syringes.	• Ensure the PV conductance catheter has all sensing electrodes within LV or RV. If all segments are not active adjust the catheter position until all segments are active. Sometimes a slight twisting of the catheter may be necessary to optimize loop morphology • Pressure data were digitally acquired continuously whereas volume was recoded at 1-mL intervals to obtain an optimal configuration. • Ensure the contralateral ventricle is empty when PV measurements are performed in the selected chamber.	N=8	Yes
