# Supplementary figures and images for: Data from acellular human heart matrix
Source: Data Brief. 2016 May 18;8:211–9. doi: 10.1016/j.dib.2016.04.069 (PMC4900585; doi:10.1016/j.dib.2016.04.069)

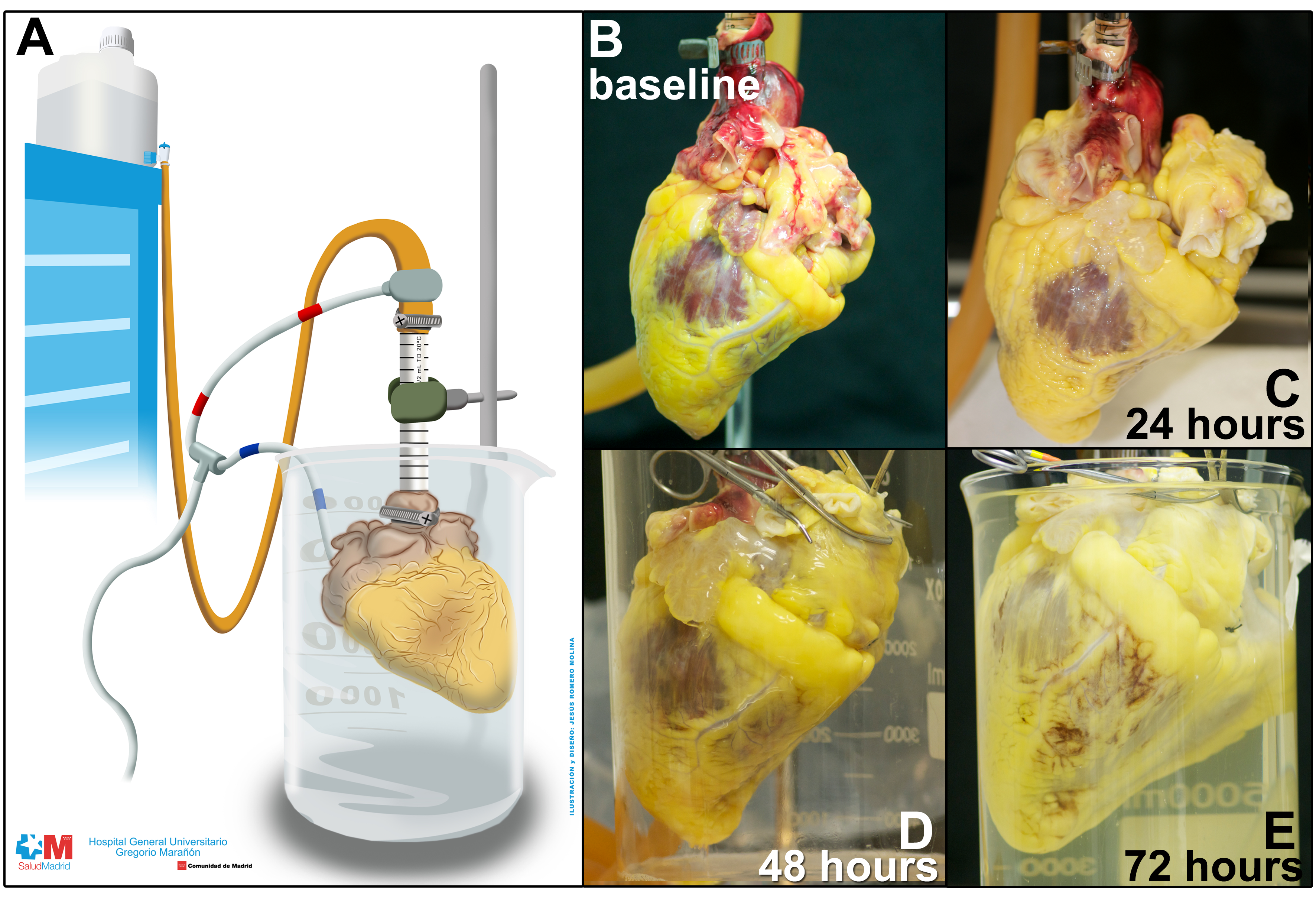

Supplement: Supplementary file 2 — Supplementary material [file mmc2.zip › Supplementary Figure 1.tif]

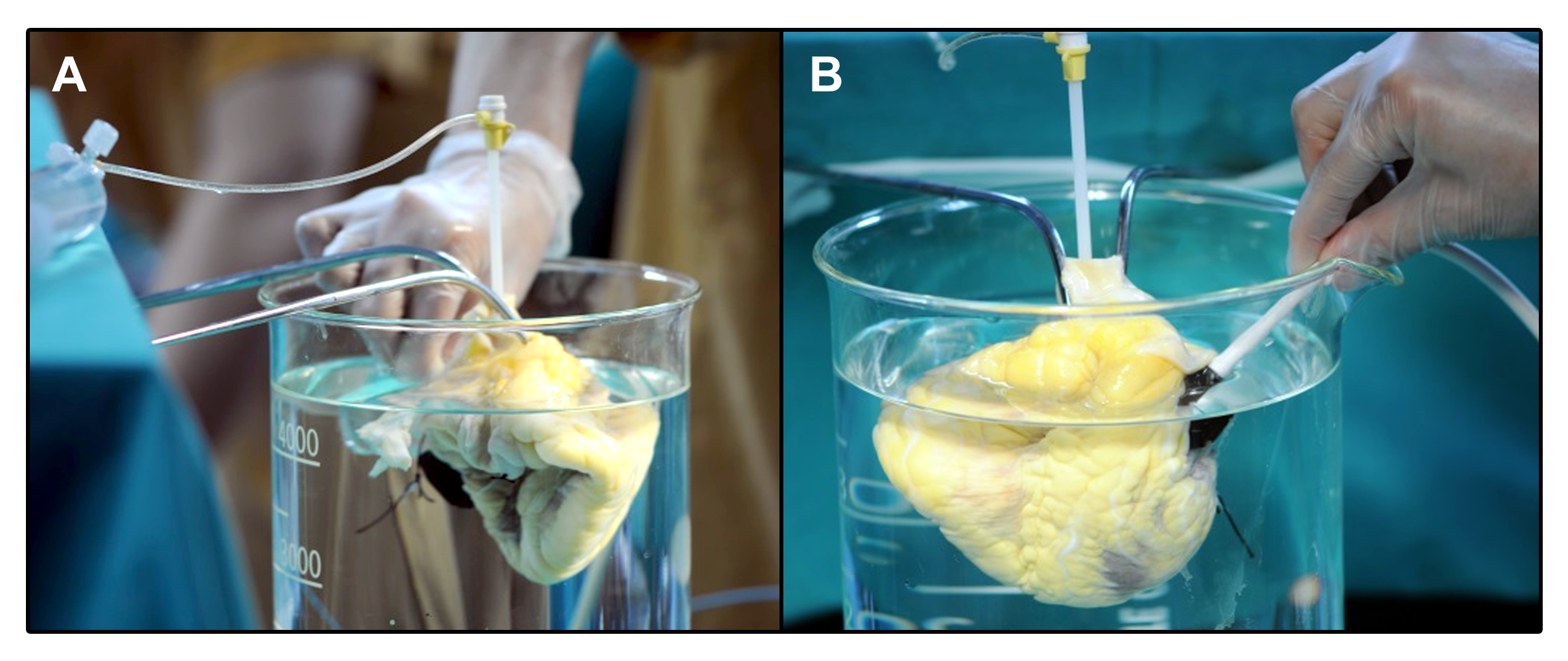

Supplement: Supplementary file 3 — Supplementary material [file mmc3.zip › Supplementary Figure 2.tif]

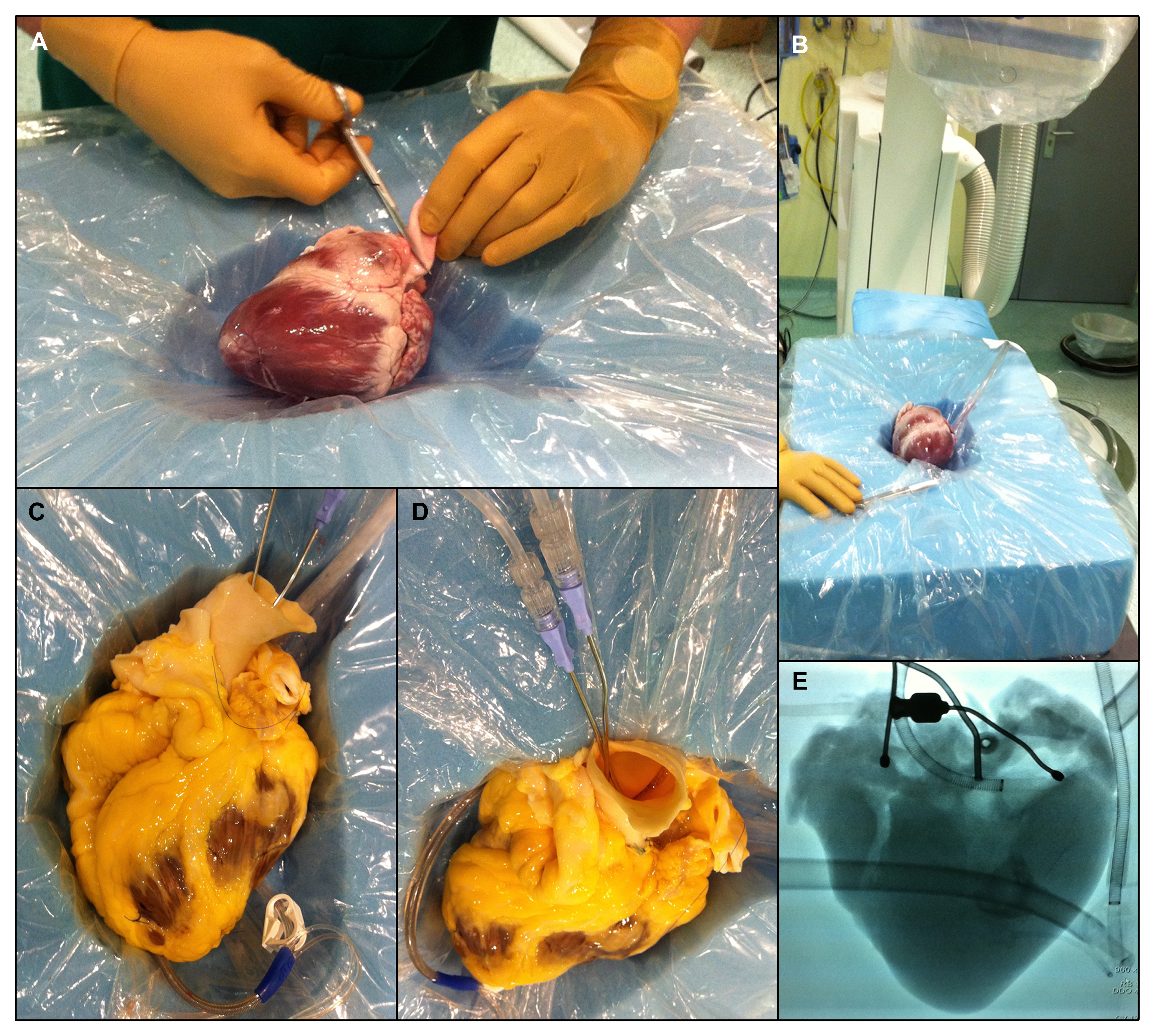

Supplement: Supplementary file 4 — Supplementary material [file mmc4.zip › Supplementary Figure 3.tif]

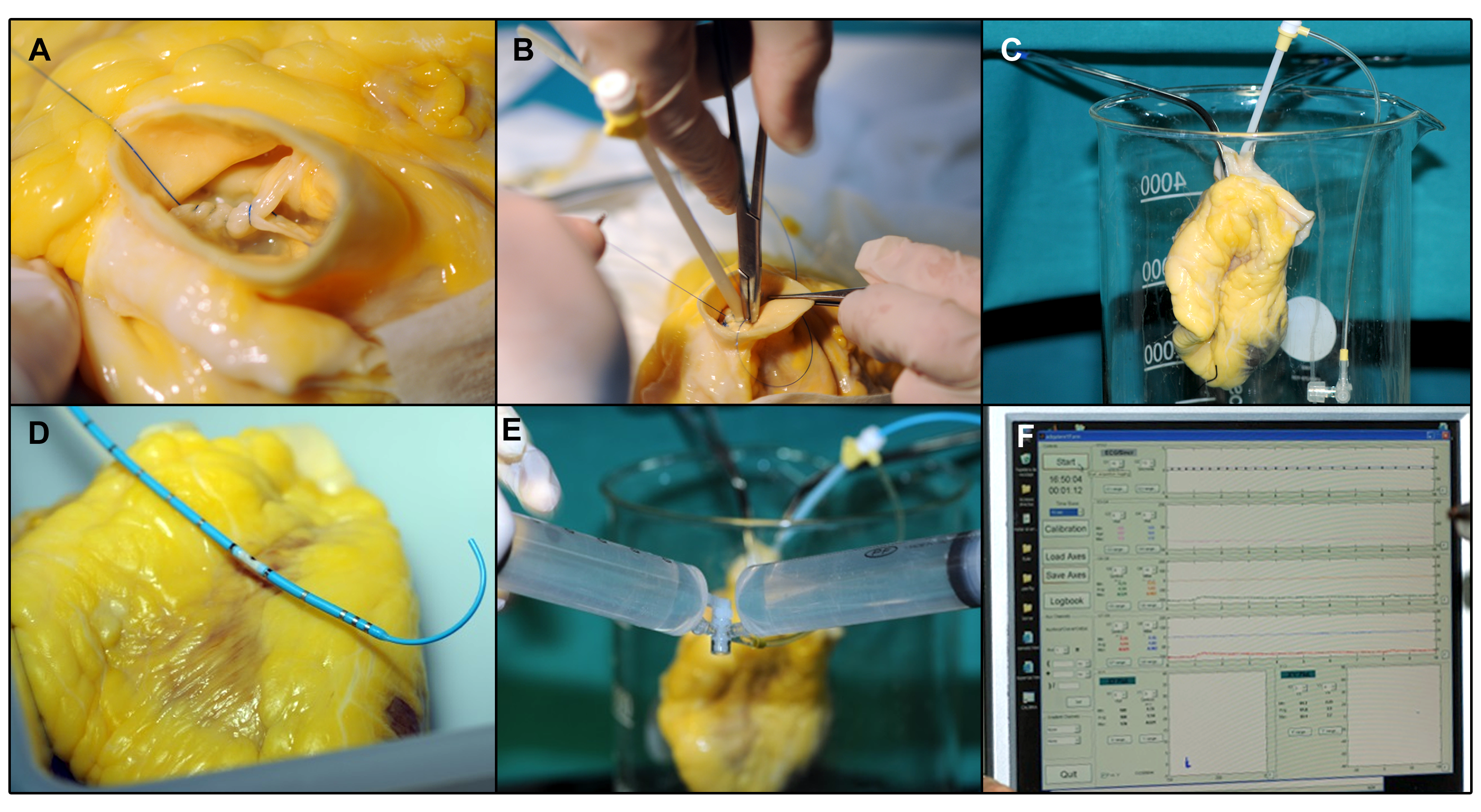

Supplement: Supplementary file 5 — Supplementary material [file mmc5.zip › Supplementary Figure 4.tif]
