# Supplementary material for: Data from acellular human heart matrix
Source: Data Brief. 2016 May 18;8:211–9. doi: 10.1016/j.dib.2016.04.069 (PMC4900585; doi:10.1016/j.dib.2016.04.069)
Supplement: Supplementary file 1 — Supplementary material [file mmc1.doc]

Prof. Hao-Ran Wang, PhD

Editor-in-Chief

Data in Brief

Re: conflict of interest

Dear Prof. Wang,

Thank you very much for your review of our manuscript DIB-D-15-00110.

I certify I don’t have any conflict of interest to disclose

We thank you in advance for all the work involved in reviewing this manuscript and for the opportunity to submit it again for your consideration.

We look forward to your future correspondence.

Sincerely,

Francisco Fernández-Avilés
